# Harmonized Database of Western U.S. Water Rights (HarDWR) v.1

**DOI:** 10.1038/s41597-024-03434-6

**Published:** 2024-06-06

**Authors:** Matthew D. Lisk, Danielle S. Grogan, Shan Zuidema, Jiameng Zheng, Robert Caccese, Darrah Peklak, Karen Fisher-Vanden, Richard B. Lammers, Sheila M. Olmstead, Lara Fowler

**Affiliations:** 1 Pennsylvania State University, Earth and Environmental Systems Institute, University Park, PA USA; 2 Earth Systems Research Center, Institute for the Study of Earth, Oceans, and Space, University of New Hampshire, Durham, NH USA; 3 University of Illinois at Urbana-Champaign, Gies College of Business, Urbana, IL USA; 4 Director of Policy, Planning and Communications, Pennsylvania Fish and Boat Commission, Harrisburg, USA; 5 Moody’s Analytics, New York, USA; 6 Pennsylvania State University, Agricultural Economics, Sociology, and Education, University Park, PA USA; 7 University of Texas at Austin, LBJ School of Public Affairs, Austin, TX USA; 8 Resources for the Future, Washington, DC USA; 9 Property and Environment Research Center, Bozeman, MT USA; 10 Pennsylvania State University, Penn State Law, University Park, PA USA

**Keywords:** Water resources, Economics, Interdisciplinary studies

## Abstract

In the arid and semi-arid Western U.S., access to water is regulated through a legal system of water rights. Individuals, companies, organizations, municipalities, and tribal entities have documents that declare their water rights. State water regulatory agencies collate and maintain these records, which can be used in legal disputes over access to water. While these records are publicly available data in all Western U.S. states, the data have not yet been readily available in digital form from all states. Furthermore, there are many differences in data format, terminology, and definitions between state water regulatory agencies. Here, we have collected water rights data from 11 Western U.S. state agencies, harmonized terminology and use definitions, formatted them for consistency, and tied them to a Western U.S.-wide shapefile of water administrative boundaries.

## Background & Summary

Water scarcity is a challenge in arid regions across the world^1^ and is managed by a wide range of governance and institutional approaches^2,3^. As climate change and competition for water between uses continues to add pressure to already water-stressed regions^4,5^, managers, policy makers, and scientists are seeking alternative management strategies to the insufficient policies currently in place^3^. One such region is the Western U.S., where water stress has increased due to several factors including long-term drought^6^, increasing competition between agricultural and urban water users^4^, and new valuation of in-stream flows^7^.

The arid Western U.S. began regulating water allocations during the gold rush period of the mid 1800’s^8^. During this time, water was essential for mining, and so the Prior Appropriation Doctrine for water allocation – which is largely still in use today – grew out of gold mining’s system of prioritizing resource allocation based on the date when an individual or organization first laid a claim. This is known as “first in time, first in right”, and establishes a system of seniority for water users. Following this 1855 ruling in California, many other Western U.S. states established their own forms of water regulation based in part or in whole on the Prior Appropriation Doctrine. We refer readers to refs. ^9,10^ for a thorough history of the Prior Appropriation Doctrine generally in the U.S. West, to refs. ^11,12^ for a history of California’s water regulatory system, ref. ^13^ for discussion of water governance in New Mexico, and ref. ^14^ for a review of the impact of pre-colonial Spanish rights on the region.

More recently, states and water regulatory institutions have modified water laws and adjusted implementation of the Prior Appropriation Doctrine in a variety of ways^9^. Spatial variation in these modifications between states and even within states has led to a patchwork of water rights systems. Further, some Western states regulate groundwater with the same Prior Appropriation Doctrine as surface water, while others do not. This legal dichotomy between groundwater and surface water causes practical management challenges. In many jurisdictions, surface and groundwater systems are highly interdependent and conjunctive management strategies are made more difficult when groups of users are assigned allocations by either groundwater sources or surface water sources^15^. Lastly, multiple but not all water administrative units have adjudicated their water rights. These include, but are not limited to, the Snake River Basin Adjudication in Idaho^10^ and multiple basins across New Mexico^13^ and California, and more recently multiple groundwater basins in California^16^. Adjudication is the process of accounting for all water rights holdings and resolving any disputes or conflicts between water rights holders. Lack of adjudication across much of the Western U.S. means that many water rights documents lay claim to water that may not be available in the river basin, leading to over-allocation of water resources^17^.

Extensive criticisms and identification of the many obvious and more subtle problems of applying the Prior Appropriation Doctrine to modern water management have been written^2,18–22^. In addition to the problems of over-allocation and the patchwork legal system, this doctrine fails to reflect current values of water, for example ecological flows, urban vs. agricultural water needs, and equitable resource management.

To understand the implications of the current water rights system and evaluate solutions to its many challenges, researchers require usable water rights data for analysis^23^. Water rights data are used in the fields of basic hydrology^24^, applied water resources management and conservation^25^, climate change impacts^20,21^, and natural resource economics^26^. In the US West water rights databases have been available only from individual states, either by accessing publicly available digital databases or by contacting state water regulatory agencies and requesting the data. However, states’ data formats, water use categories, and identification systems differ, making region-wide analysis difficult. Cross-state data consistency is expected to help analysis of water needs and shortfalls across the region.

Here we present a new database of Western U.S. water rights records^27–30^. This database provides consistent unique identifiers for each spatial unit of water management across the domain, unique identifiers for each water right record, and a consistent categorization scheme that puts each water right record into one of 7 broad use categories. These data were instrumental in conducting a study of the multi-sector dynamics of intersectoral water allocation changes through water markets^31^. Specifically, the data were formatted for use as input to a process-based hydrologic model, the Water Balance Model (WBM)^32^, with a water rights module^31^. While this specific study motivated the development of the database presented here, U.S. Western water management is a rich area of study^17,21,23,33^, so we hope that releasing this database publicly with documentation and usage notes will enable other researchers to do further work on water management in the U.S. West. We also hope that sharing this database will lead to improvements as the research community finds ways to enhance, correct, or adjust this database.

## Methods

### Overview

We produced the water rights database presented here in 4 main steps, each described in detail below: (1) data collection, (2) data quality control, (3) data harmonization, and (4) generation of cumulative water rights curves. Each of steps (1) - (3) had to be completed to produce (4), the final product that was used in the modeling exercise in ref. ^31^. All data in each step is associated with a spatial unit called a Water Management Area (WMA), which is the unit of water right administration. Steps (2) and (3) required us to make assumptions and interpretations, and to remove records from the raw data collection. We describe each of these assumptions and interpretations below, and provide products resulting from steps (1), (3), and (4) so that other researchers can choose to implement alternative assumptions and interpretations as fits their research aims. We also describe all data sources used in step (1) and provide all code used in steps (2) - (4) so that others may follow our methods with updated data, as water rights databases from the states are not static but rather change over time. Note that one piece of code was used to do all work described in steps (2) and (3); their distinction is for the purposes of description here.

1. **Data collection**
  To hold a water right in the Western U.S., an entity, (e.g., an individual, corporation, irrigation district, municipality, sovereign government, or non-profit) must register a physical document with the state’s water regulatory agency. State water agencies each maintain their own database containing all registered water right documents within the state, along with relevant metadata such as the point of diversion (which is the location of water extraction) and place of use of the water. All Western U.S. states have digitized their individual water rights databases, along with the geospatial data describing the spatial units where water rights are managed. Each state maintains and provides their own water rights data in accordance with individual state regulations and standards.
  We collected water rights databases from 11 Western U.S. states either by downloading them from publicly accessible web portals, or by contacting state water management representatives. Supplementary Table 1 reports the water rights data sources used for each state. Supplementary Table 2 reports the data sources we used to delineate water management area (WMA) spatial boundaries by each state. Note that these data were collected over a period of 4 years, and that many states regularly update their databases on a yearly, monthly, and even daily schedule as new water rights are registered, existing water rights are traded, and old water rights become inactive.
  This step produced data product (1) Raw Data, described below in the Data Records section.
2. **Data quality control**

Our aim in the quality control step was to remove entries from the raw water rights database that were unusable in our analysis due to missing essential information, obviously erroneous information, or duplication of entries. In some cases, we were able to correct erroneous information such as spelling inconsistencies in character strings. All quality control steps were done in the programming language *R*.

#### *Region-wide quality control*

Common reasons for removing records, and the number of records removed from each state’s raw database for each reason are given in Table 1.

**Table 1:** For each state, we report the number of records in the raw database and the total # of records removed in the quality control step. The number of records removed for each of seven common issues is reported as well.

State	# Raw Records	Total # Rights Removed	# Invalid Right ID	# Duplicates	# Invalid Flow/Volume	# Invalid Water Source	# Invalid Priority Date	# Invalid Basin	# Invalid Water Use
Idaho	289704	82925	12093	70813	0	0	19	0	0
Washington	37328	12534	3	2960	0	0	8278	13	1280
Oregon	189155	114	0	0	0	0	114	0	0
California	359495	308922*	0	49158	256818	5	0	1644	43
Colorado	187888	6480	0	6336	142	0	0	0	2
Arizona - Well Registry	212019	209802*	207441	486	68	0	1807	0	0
Arizona - Surface Water Claims	215001	85535	0	65460	10151	0	8003	1109	812
Arizona - Statement of Claimant	125485	103438*	0	0	99906	1465	2067	0	0
Nevada	28085	743*	0	0	0	0	743	0	0
Utah	512904	44246**	78	0	88	0	30580	0	13500
New Mexico	237427	79915	0	0	38	0	79877	0	0
Montana	2368941	2085423*	0	1409079	181290	21	30715	0	464318
Wyoming	266796	73656*	0	1911	45784	0	69	0	25892

^*^Records were added/copied when data was joined.

^**^There are known record duplicates with unique location coordinates.

Entries were removed from each database if they were missing information (either blank, or “NA” value) in any of the following fields: a priority date, a water source (e.g., surface or groundwater), and water use category (e.g., irrigation), a volume or flow of water allocated and an associated recognizable unit (e.g., cubic feet per second) and a water management area. Different states’ databases had different column headers for these pieces of information, but all state databases contained these essential informational fields in some way. Many state databases contain additional fields of information. Entries were not removed for missing data in any of the additional fields.

Each state database uses its own unique identifier field for water rights records. We found many state databases containing multiple entries with the same unique identifier. In this case, only one entry was kept, while the others were removed from the database. If the water flow or volume allocations of the duplicate entries were identical, then we kept the first entry. If the allocations differed, then we kept the entry with the largest allocation.

All state databases contain a water source field. We categorized “groundwater”, “ground water”, “GW”, “OGW”, “Underground”, “well”, and “abandoned well” character strings in this field as groundwater. We then assumed that every entry not identified as groundwater was sourced from surface water. This allowed us to avoid encoding the many water source character strings used to describe surface water sources (e.g., specific river, stream, reservoir, or canal names).

Many state databases contain a spatial coordinate and a field listing the WMA associated with each record. In cases where the spatial coordinates fell outside of the record’s stated WMA, we chose to keep the WMA value and discard the spatial coordinates.

#### *State-specific quality control*

In addition to the four quality control steps described above, we found that each state’s data had unique characteristics that required unique solutions to make the database usable for our analysis. Here we report these state-by-state assumptions. The code for these steps is publicly available and described in the Code Availability section below. We report in detail the modifications and assumptions applied partly to show the type of assumptions we had to make, which reveals it is likely that further corrections would improve the database. For example, we had to correct spelling errors and remove double spaces in some character strings to ensure consistent merging of water rights records and spatial data. We found these required corrections by examining each state’s database visually.

##### Idaho

We processed the water rights database from the state of Idaho first for two reasons: (i) We were able to work with local experts in Idaho including state water resources managers for several years, helping us to understand how water rights are claimed, data are collated, and laws are implemented; this provided a foundation for the database development for the entire region; (ii) water rights in the Snake River basin of Idaho have been adjudicated, providing an ideal region for initial data and model testing as this ensured a high level of consistency and transparency in the water rights data. In Idaho, we obtained water rights data from 2 different sources. The first source is the publicly available database from the IDWR (Supplementary Table 2). However, this database has “NA” values in the Water Use Code column for many entries. We found that a large portion of these entries were for rights held by the federal government. By contacting the IDWR directly, we were able to obtain supplementary data that filled in the federal water rights use data. We merged these two databases to provide a more complete final product for Idaho. We assumed that water source character strings “Underground” and “Abandoned Well” should be categorized as groundwater sources.

##### Washington

Washington state provides water rights data as a set of 3 files, two spatial feature classes and one text file. One spatial feature file is a point of diversion (POD), and the other is a place of use (POU). The text file provides the water use associated with each POU. We merged these three files by the state’s unique identifier field.

##### Oregon

Oregon provides its water rights data in 2 files, a point of diversion and a place of use shapefile. We found that the place of use files contained no data readable in R, so we were only able to use the point of diversion files.

##### California

California point of diversion data are provided as Excel® spreadsheet files and are bundled as separate collections of files for each county, for each water use, for each entity, and for each watershed. While theoretically all water rights records should be present in each collection, we found differences between the collections. We loaded all collections into *R* and merged them to create the maximum set of all water rights records. In this merged database we corrected issues with character strings to enable merging of water rights entries with spatial data. These corrections were performed by visual inspection of the database. Specifically, we removed double spaces in character strings of the WMAs, for example changing “Santa  Maria” to “Santa Maria”, and corrected occasional spelling errors such as “Bolsa N**eu**va” to “Bolsa N**ue**va”.

##### Colorado

Colorado provides water rights as a set of point of diversion text files, organized by district. The database has both geographic (latitude, longitude) and Universal Transverse Mercator (UTM) coordinates, but we found that the UTM coordinates were often outside the reported WMA and in some cases even outside the bounds of the Western U.S. Therefore, we only used and retained the geographic coordinates on our merged database.

##### Arizona

Arizona regulates water in a different way than the other 10 states. Outside of some relatively small critical agricultural areas called Active Management Areas (AMAs), Arizona does not maintain any water rights. However, the state does require registration of surface and groundwater pumping devices, which includes disclosing the mechanical specifics of the devices. We used these records as a proxy for water rights, with the maximum pumping capability used as equivalent to the annual water allowed by the right, install date as equivalent to priority date, and equipment location as the equivalent to point of diversion. Groundwater is only regulated in a subset of all Active Management Areas and done so via well registries. The well registry data is provided as two collections of text files: well registry forms that provide the location of the wells, and Statement of Claimant data that provide the use, water source, priority date, and all other relevant data. Surface water extraction locations are provided as spatial shapefile, as well as coordinates listed in the Statement of Claimant data. We merged the groundwater and surface water entries into a single spatial data frame. Some entries had missing data in the water rights management area field but contained spatial coordinates. We intersected the spatial coordinates of the record with the WMA shapefile for Arizona to fill in this missing information. In some cases, the two datasets provided differing coordinates for a record. In these cases, we selected the mean center between points identified in the two data sources.

##### Nevada

Nevada provides point of diversion shapefiles for their water rights. Only the region-wide quality control steps needed to be applied here.

##### Utah

Utah provides point of diversion and place of use shapefiles for their water rights. We only used the point of diversion, as the place of use data was missing required information including water right identifying information that allows merging with the point of diversion. Some entries include a unique identifier character string “-”; these were treated as missing data and removed from the database. Some priority date entries contain dates that don’t exist, typically identifying the day as the 31st of a month in which there is no 31st day (e.g., February 31). These dates were changed to the actual last day of the month.

##### New Mexico

New Mexico provides point of diversion shapefiles for their water rights. This data contains date fields for “start_date” and “finish_dat”. We only removed records with missing data in both fields; records with one date or the other entered were kept, and that single date was used as the priority date. Where both dates are provided, we used the “start_date” field as the priority date.

##### Montana

Montana provides their water rights as two geodatabases (.gdb files), one point of diversion and one place of use. Some records in these databases have more than one water use identified. In these cases, we used and kept only the first water use listed.

##### Wyoming

Wyoming water rights data are provided as text files. We found a character string inconsistency between the WMA field in the text files and the geospatial data containing the WMA boundaries, so we changed the character string “1_15” to “1_15-5” in the water management area field of the water rights records. As with the Montana data, some records have more than one water use identified. In these cases, we used and kept only the first water use listed.

The result of this data quality control was a collection of 11 state water right databases, each containing only records with the required information and with as many corrections to unique issues as we could identify. The next step, data harmonization, brought these individual databases together.

(3) **Data harmonization**

The purpose of data harmonization was to take the quality-controlled, individual state water rights databases and turn them into a single, consistent database. The following steps were applied to all 11 state databases for this purpose:

- Each record was given a unique identifier. The state-specific unique identifier was retained as an entry field, but this new identifier solves the issue of multiple state records having the same state-specific identifiers (e.g., record 1101).
- Each WMA was given a unique identifier.
- Water allocations reported in volume were converted to flows in cubic feet per second, assuming that the volume is an annual flux value.
- Water allocations reported in flow units other than cubic feet per second were converted to cubic feet per second.
- Priority date values were all converted to formats of YYYY/MM/DD

To combine the water rights data across states, we developed 7 broad water-use categories: Irrigation, Domestic, Livestock, Fish, Industrial, Environmental, Other. We then used each state’s classification descriptions, expert opinion, and local knowledge to map each record’s state classification to these 7 categories. Supplemental Table 3 lists all character strings from water use fields in the combined 11-state water rights database grouped into each of the 7 broad categories. Note that the character strings in the state databases are reported here without any changes, so that spelling errors or extra spaces are retained in Supplemental Table 3.

The states each report the spatial extent over which water rights are managed. The names for management units are unique to each state, but here we adopt the term water management area (WMA). The areal extent of these units is concatenated from the original sources into a single multi-polygon shapefile and given common field names. No other actions are taken to modify the shapefiles, which can lead to artifacts of inconsistent WMA boundaries particularly along state boundaries that users of the data product should be aware of when interpreting analyses using these data.

(4) **Creating cumulative water rights curves**

Our last processing step was to generate a set of input data for the hydrologic model, WBM, using the quality-controlled, harmonized 11-state database described above. In addition to the required fields of priority date, use category, water management area, water source, and flow allocation, WBM also requires information on the total, cumulative water flow that has been allocated over time. The model’s water rights module then distributes water between use categories within each WMA based on the proportional distribution of water rights to each category for a given cumulative flow.

To calculate the cumulative water rights, we first gap-filled the list of priority dates for each WMA from 217 CE to 2100. These dates are the earliest and latest priority dates found across the entire database. While these end-member dates are difficult to interpret, we assumed all priority dates were accurate as reported, and instead incorporated all listed interpretable dates into the cumulative curves. Any WMAs without any new water rights allocations in a year between 217 CE and 2100 were given a 0 ft^3^s^−1^ new flow allocation for that year. Very few rights were allocated prior to the 1800s, so most cumulative water rights curves have a long sequence of 0 flow allocations at the beginning. Then, for each priority date year, we summed all allocated flows across use categories to calculate the total flow allocation for that priority date. Then we calculated the cumulative sum for each priority date, both for the total flow allocation and for each use category’s allocation. Lastly, we calculated the proportion of total cumulative allocations assigned to each use category in each priority date year. We repeated this process for each WMA and for surface water separately from groundwater. Not all states regulate (or did regulate, prior to 2020) groundwater with water rights allocations in the same way as surface water. In the case of a WMA with surface water rights but no groundwater rights, we generated a cumulative groundwater rights curve file with all values for flow allocations at 0 ft^3^s^−1^ because the model WBM requires both a surface water and a groundwater cumulative right input file for each WMA.

## Data Records

There are four data products associated with this work: Raw water rights data^27^, Harmonized water rights data^28^, Cumulative water rights curves^29^, and the Water Management Area shapefile^30^.

(A) Raw water rights data^27^
  We provide the raw water rights data collected from the water regulatory agencies for 11 states, as described in Supplementary Table 1. Each state formats their data differently, meaning that file types, field availability, and names vary from state to state. Note, the data provided here reflects the state of the water rights databases at the time we collected the data; updates have likely occurred in many states. Some pieces of information are common among all states. These are: priority date, volume or flow of water allowed by the right, stated water use of the right, and some means of identifying the geography and source of the water pertaining to the right - typically the coordinates of the Point of Diversion (PoD) of a waterbody or well.
  The available data is provided as a series of compressed files, each containing the full data collected from each state. Some of the files have been renamed, to more easily know which state the data belongs to. The file renaming was also required as some files from different states had the same name. In other cases, the data for a state has been placed in a folder indicating which state it belongs to - as the state organized its data by selected subregions. Below is a brief description of the format of the collected data from each state.
  **ArizonaRights_StatementOfClaimants** - A folder containing a database of interconnected CSV files. The soc_erd.pdf file contains a visual flowchart of how the various files are connected, beginning with SOC_MAIN.csv in the center of the page.
  **ArizonaRights_SurfaceWaterRightsData** - A folder containing a database of a single Shapefile and 10 associated CSVs. SurfaceWater.pdf contains a visual flowchart of how the various files are connected, beginning with ADWR_SW_APPL_REGRY.csv.
  **ArizonaRights_Well55Registry** - A folder containing a database of a single Shapefile and 59 associated CSVs. Wells55.pdf contains a visual flowchart of how the various files are connected, beginning with WellRegistry.shp.
  **CaliforniaRights_eWRIMS_directDatabase** - A folder containing a collection of four “series” Microsoft Excel files, as either XLS or XLSX. The four “series”: byCounty, byEntity (what type of legal entity holds the right), byUse (stated water use), and byWatershed, are various methods by which the California water rights are organized within the state’s database. However, it was observed that by only collecting a single series, not all water rights were being provided. So, essentially, most records within each “series” are copies of each other, with each “series” containing some unique records.
  **ColoradoRights_NetAmounts** - A folder containing 78 CSV files, with one file per Colorado Water District.
  **IdahoRights_PointOfDiversion** - A Shapefile containing the Points of Diversion for the entire state of Idaho.
  **IdahoRights_PlaceOfUse** - A Shapefile containing the Place of Use polygons for the entire state of Idaho.
  **MontanaRights_WaterRights** - A Geodatabase file containing the Points of Diversion and Places of Use for the entire state of Montana. The name of the Points of Diversion Feature Layer within the Geodatabase is “WRDIV”, and the name of the Places of Use Feature Layer is “WRPOU”.
  **NevadaRights_POD_Sites** - A Shapefile containing the Points of Diversion for the entire state of Nevada.
  **NewMexicoRights_Points_of_Diversion** - A Shapefile containing the Points of Diversion for the entire state of New Mexico.
  **OregonRights_state_shp** - A folder containing 36 Shapefiles and are split between “pod” (Point of Diversion) and “pou” (Place of Use) for each water management basin within Oregon. In other words, each basin has one “pod” file and one “pou” file. The “pod” files are point shapes, and the “pou” files are polygons.
  **UtahRights_Points_of_Diversion** - A Shapefile containing the Points of Diversion for the entire state of Utah.
  **WashingtonRights_WaterDiversions_ECY_NHD** - A Geodatabase file containing both the Points of Diversion for the entire state of Washington. The name of the Feature Layer within the Geodatabase is “WaterDiversions_ECY_NHD”.
  **WyomingRights** - A folder containing four subdirectories, one for each Wyoming Water Division. Each Division directory includes a varying number of subdirectories for each Wyoming Water District. Each District folder contains two copies of the Point of Diversion records for that area, with one copying being in CSV and one copy in Microsoft Excel XLS format.
  **Data** 10.57931/2004664
  **Available from:** msdlive.org
(B) Harmonized water rights data^28^
  The dataset is a series of various files organized by state sub-directories. The first two characters of each file name is the abbreviation for the state in which the file contains data for. After the abbreviation is the text which describes the contents of the file. Here is each file type described in detail:
  **XXFullHarmonizedRights.csv**: A file of the combined groundwater and surface water records for each state. Essentially, this file is the merging of XXGroundwaterHarmonizedRights.csv and XXSurfaceWaterHarmonizedRights.csv by state. The column headers for each of this type of file are:
  *state* - The name of the state the data comes from.
  *FIPS* - The two-digit numeric state ID code.
  *waterRightID* - The unique identifying ID of the water right, the same identifier as its state uses.
  *priorityDate* - The priority date associated with the right.
  *origWaterUse* - The original stated water use(s) from the state.
  *waterUse* - The water use category under the unified use categories established here.
  *source* - Whether the right is for surface water or groundwater.
  *basinNum* - The alpha-numeric identifier of the WMA the record belongs to.
  *CFS* - The maximum flow of the allocation in cubic feet per second (ft3s-1).
  Arizona is unique among the states, as its surface and groundwater resources are managed with two different sets of boundaries. So, for Arizona, the basinNum column is missing and instead there are two columns:
  *surBasinNum* - The alpha-numeric identifier of the surface water WMA the record belongs to.
  *grdBasinNum* - The alpha-numeric identifier of the groundwater WMA the record belongs to.
  **XXStatePOD.shp:** A shapefile which identifies the location of the Points of Diversion for the state’s water rights. It should be noted that not all water right records in XXFullHarmonizedRights.csv have coordinates, and therefore may be missing from this file.
  **XXStatePOU.shp:** A shapefile which contains the area(s) in which each water right is claimed to be used. *Currently, only Idaho and Washington provided valid data to include within this file*.
  **XXGroundwaterHarmonizedRights.csv:** A file which contains only harmonized groundwater rights collected from each state. See XXFullHarmonizedRights.csv for more details on how the data is formatted.
  **XXSurfaceWaterHarmonizedRights.csv:** A file which contains only harmonized surface water rights collected from each state. See XXFullHarmonizedRights.csv for more details on how the data is formatted.
  Additionally, one file, **stateWMALabels.csv**, is not stored within a sub-directory. While we have referred to the spatial boundaries that each state uses to manage its water resources as WMAs, this term is not shared across all states. This file lists the proper name for each boundary set, by state.
  **Database size:** 357.1 MB uncompressed
  **Data** 10.57931/2341234
  **Available from:** msdlive.org
(C) Cumulative water rights curves^29^
  This product is the database used as input to the WBM model and is the result of step (4) *Creating cumulative water rights curves* described above.
  **File naming convention:** WMA_[###]_[X]W.csv, where [###] is the unique identifier for each WMA, and [X] is either S for surface water rights, or G for groundwater rights.
  **Average single file size:** 74 K
  **Total database size:** 138 M
  **Column headers in each file:**
  **Year:** the priority date year
  **CUML:** the total cumulative water rights allocated up to this priority date year (ft^3^s^−1^)
  **Irrigation:** The percent of cumulative water rights allocated to the Irrigation category up to this priority date year (%)
  **Domestic:** The percent of cumulative water rights allocated to the Domestic category up to this priority date year (%)
  **Livestock:** The percent of cumulative water rights allocated to the Livestock category up to this priority date year (%)
  **Fish:** The percent of cumulative water rights allocated to the Fish category up to this priority date year (%)
  **Industrial:** The percent of cumulative water rights allocated to the Industrial category up to this priority date year (%)
  **Environmental:** The percent of cumulative water rights allocated to the Environmental category up to this priority date year (%)
  **Other:** The percent of cumulative water rights allocated to the Other category up to this priority date year (%)
  In addition to the database files, there is a companion.csv file, called stateWMAs_ID.csv. The main purpose of this file is to provide the means of translating between the ### unique identifier and the various other id of the WMA the file is attached to. This translation file has five columns, which are:
  **basinNum**: The official state given alpha-numeric identifier of the WMA.
  **basinName**: the state provided English name of the area, where applicable
  **state**: the state name
  **uniID**: a unique identifier we created by concatenating the state name, and underscore, and the state numerical ID.
  **ID**: a unique numeric identifier we created as a requirement for the files to be used within WBM (Grogan *et al*., *in review*).
  **Data:** 10.57931/2325001
  **Available from:** msdlive.org
  Tables 2, 3 show the kind of regional summaries that are easy to extract from the cumulative water rights curves.

**Table 2:** Summary of surface and groundwater rights by state included in the cumulative water rights curves (Data Product 3).

State	Total state allocations in 1000 ft^3^ s^−1^	
	Surface Water	Groundwater
Arizona	75,706*	352**
California	3,065	***
Colorado	360	37
Idaho	249	42
Montana	17,046	29
Nevada	34	14
NewMexico	3	18
Oregon	1,519	47
Utah	1,574	3,426
Washington	1,606	0
Wyoming	911	21

*In Arizona, this number is the sum of permitted conveyances and flow rates.

**In Arizona, this number is the sum of pumping well registry flows.

***Prior to 2020, California did not regulate groundwater with water rights.

**Table 3:** Summary of surface and groundwater rights by sector included in the cumulative water rights curves (Data Product 3).

Sector	Total sector allocations in 1000 ft^3^ s^−1^	
	Surface Water	Groundwater
Domestic	17,434	254
Livestock	18,038	9
Other	5,095	2,910
Environmental	5,132	3
Industrial	21,640	595
Irrigation	21,574	215
Fish	13,158	2
(D) Water Management Area shapefile^30^

**Fig. 1:**
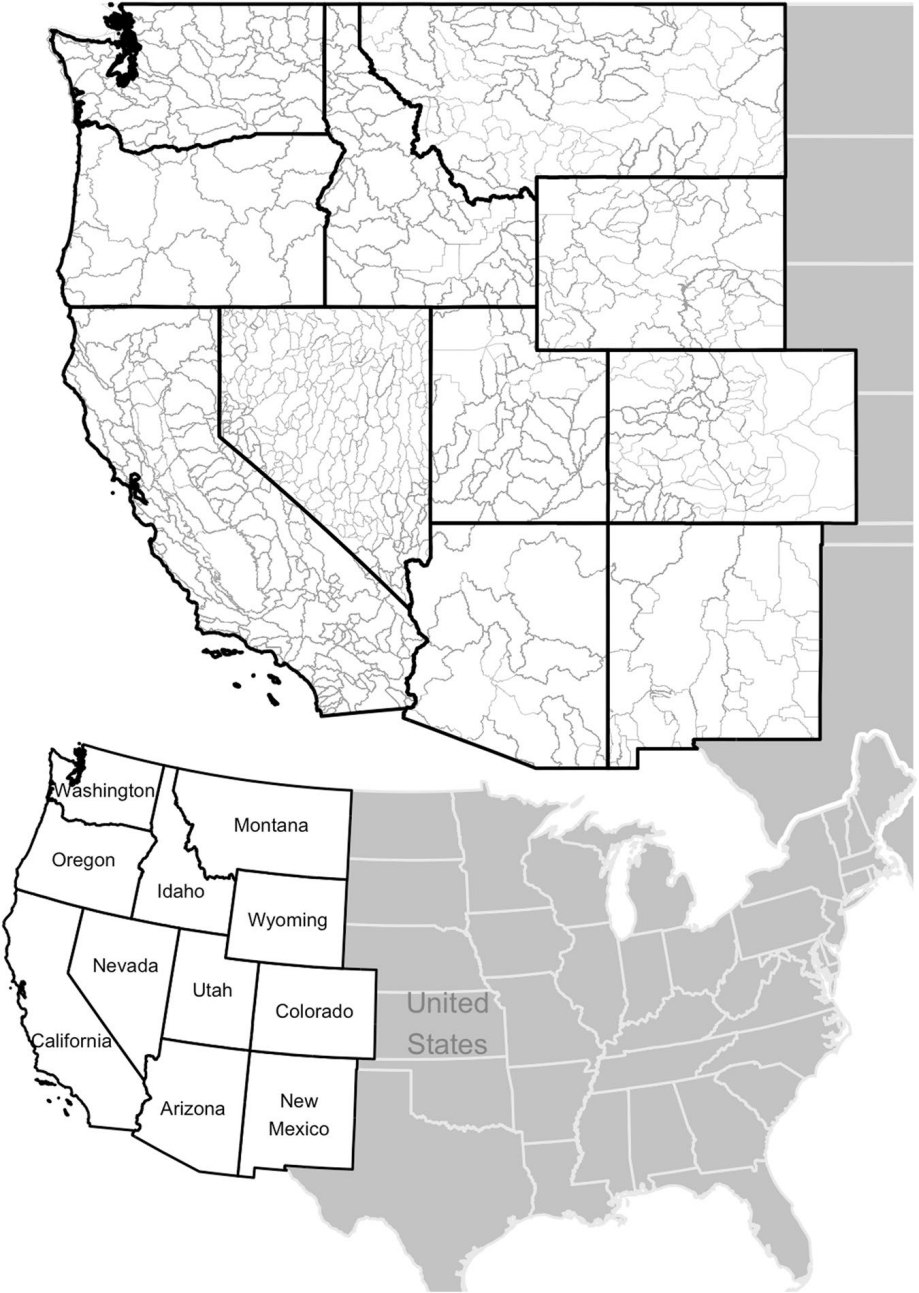
The borders of all Water Management Areas (WMAs) across the 11-state region are now available in one unified shapefile, with unique identifiers for each polygon.

Borders of all WMAs are available filtered through a single source. The data are provided as two compressed shapefiles. One contains data for all 11 states. For 10 of those states, Arizona being the exception, the polygons represent the legal management boundaries used by those states to manage their surface and groundwater resources respectively (Fig. 1). The shapes of WMAs are unaltered from the original data sources and merged into a single shapefile and given common field designations.

Arizona is unique among this collection of states in that surface and groundwater resources are managed using two separate sets of boundaries. During our follow-up analysis, due to technical reasons we decided to focus on one set of boundaries, those for surface water. Due to this, the Arizona surface WMAs are included in the above-described file. The Arizona groundwater WMAs are provided as the second file as a companion to the first file for completeness and general reference.

The columns for both shapefiles are:

**basinNum**: the state provided unique numerical ID.

**basinName**: the state provided English name of the area, where applicable

**state:** the state name.

**uniID**: a unique identifier we created by concatenating the state name, and underscore, and the state numerical ID.

**Data:** 10.57931/2001052

**Available from:** msdlive.org

## Technical Validation

### Cumulative curve technical tests

The cumulative curve data provide a useful summary of geographically aggregated water rights allocations (e.g., Tables 2, 3). We provide a utility that ensures the data meets the requirements of the WBM. This python command-line utility program checkWMA_tables.py searches a directory of WMA cumulative curves and performs several specific checks on the data. The utility prints details of tables that fail checks to the screen for the user but provides no functionality to rectify problems (if any) that it encounters. The utility performs the following checks:

1. For a range of WMA_IDs listed sequentially from 1 to 909 (the number of WMAs in the 11-state database) the utility ensures that for each WMA_ID that both surface and groundwater rights are available in the directory.
  For each WMA with listed data, the utility checks for:
2. Any null, NaN, or #N/A values in either the Cumulative allocated volume (CUML), or in the percent shares to each sector.
3. Any negative values in either the Cumulative allocated volume (CUML), or in the percent shares to each water use category.
4. Any allocations that add to more than 100% of the cumulative volume. It sums the allocated shares by all water use categories and ensures that this value is less than 100.0%.
5. If any cumulative allocated volumes decline through the allocation years. The expectation is that only new water rights can be introduced over time, and not removed.

We ran this utility program on the cumulative curve data provided here. It reported no problems for the above 5 checks. It reported that no data are available for 36 of the 909 WMAs, which we verified is correct.

## Usage Notes

Our assumptions in the quality control and harmonization steps are described above. Some of these assumptions have implications for use of the final data product. In addition, we have checked through samples of data records visually to see if we could find any issues and report these issues here.

### The time frame of allocated flows

In many cases, a flow rate was provided in the water rights allocation data, but there was no information as to the time frame over which this rate was applicable. Assuming that the rates are applied to every time unit of an entire year (e.g., if the rate was in ft^3^s^−1^, applying the rate to all seconds in a year) in some cases results in very high total extraction volumes.

### Flow rates calculated from volumes

In contrast, some water rights are provided by annual volumetric allotments (e.g. acre-ft) which we assume apply to annual total allocated rights. Because we converted all water rights to the same base units of instantaneous flow in cubic feet per second, and we made no assumptions over the timeframe that the water right applies over the course of the year, these water rights may represent lower relative flow rates than those rights reported as maximum flow rates. In some cases, raw water rights records list applicable seasonal time frames, e.g., April through August, or irrigation season. However, this information was not included in the cumulative curve calculation process, and so the flow rate is permitted over the entire year. Supplementary Table 1 provides a column indicating which states and data sources provided allocation volumes or rates to better help the user interpret whether the cumulative curves in specific localities may be based on maximum instantaneous flow rates or annual allotments.

### Duplicate water right IDs

For rights with duplicate ID numbers, we only selected the entry with the highest allocated flow or volume value and used it as the only right for that ID. Hand checking a selection of entries showed that in some cases (though not all), this left out flow volumes that should have been included in the allocation.

### Multiple points of diversion

Some rights have multiple, unique entries because the right contains multiple points of diversion. We found an example where multiple points of diversion were all listed with the total flow allocation for the right, which leads to an overestimation of the allocation.

### Early priority dates

There are many water rights with priority dates preceding the establishment of the current governing system. Some of these rights may have been established when much of the study region was under Spanish/Mexican control^9,11^.

### Inactive and withdrawn rights

We found that some rights were marked as “inactive”, “withdrawn”, or with other statements indicating they are no longer in use. Our data quality control and harmonization processes did not include these pieces of information, and so rights that are no longer active are still in the database.

In addition to the assumptions we made, there are other important considerations when using this data that come from the nature of the water regulatory system itself. While we don’t provide a comprehensive history or analysis of the U.S. West’s water regulatory system here, we describe a few of the key issues that were relevant to our own interpretation and use of the data:

### Water rights adjudication

Not all rights from all states have gone through the same level of adjudication. Some states, such as Idaho, appear to be moving toward having all their water rights adjudicated. In contrast, many of the other states appear to only have adjudicated small subsets of rights after a legal dispute has been settled. This means that overallocation of water resources, contradictory claims to the same water right or water source, water right records that belong to individuals or entities who no longer exist, and errors in the raw data are all present in the raw database and therefore influence the harmonized and cumulative data products.

### Updates to state water rights databases

As mentioned above, the products provided here represent the raw water rights data as collected on the dates listed in Supplementary Table 1. Some states have updated their databases since then. Specifically, during the data collection step, many of the states were in the process of converting historical water right data into a digital format. This means that re-collecting the raw data will result in changes to the historical data prior to the dates listed in Supplementary Table 1 as well as new water rights records claimed after the collection date.

### Spatially and temporally varying enforcement of the prior appropriation doctrine

In our work with stakeholders in Idaho^34,35^, we learned two key pieces of information to keep in mind when evaluating water rights data. First, there are many legal agreements that modify the water rights system but are not reported within any states’ water right databases (e.g., the Eastern Snake Plain Aquifer Agreement, in which groundwater users agreed to reduce their water extractions to levels below their allocated flow rates). Second, different states, municipalities, and even canal companies choose to enforce, interpret, and apply the legal water rights system in different ways. Finally, it remains possible for the state or federal governments to overrule the water rights system with emergency declarations. Examples include California’s recent Sustainable Groundwater Management Act, and the current U.S. Federal Government’s Investing in America agenda which implements 8 new water conservation agreements in Arizona.

### Supplementary information

Supplementary Table 1

Supplementary Table 2

Supplementary Table 3

## Data Availability

The code used to generate the cumulative curves from the raw data is available on GitHub: https://github.com/pches/HarDWR This GitHub repository includes: *waterRightAnalysis.def* - A Singularity definition file used to create the run environment for the scripts described below. *wrDataHarmonization.R* - This script reads in the raw water rights data and performs the harmonization of the records. *wrDataHarmonization_CustomFunctions.R* - A supporting file for wrDataHarmonization.R which contains all the required custom written functions for the main script. *wrCumulation.R* - This script reads in the harmonized water rights data and performs the calculations used to create the cumulative curves. *wrCumulation_CustomFunctionsR* - A supporting file for wrCumulation.R which contains all the required custom written functions for the main script. *checkWMA_tables.py* - A script used to validate the calculated cumulative curve values. *simplifyWMAs.R* - The script which reads in the raw downloads of the various state’s WMAs as spatial data, harmonizes them, and merges them into two spatial layers.
