# Supplementary material for: Harmonized Database of Western U.S. Water Rights (HarDWR) v.1
Source: Sci Data. 2024 Jun 6;11:598. doi: 10.1038/s41597-024-03434-6 (PMC11156903; doi:10.1038/s41597-024-03434-6)
Supplement: Supplementary file 1 — Supplementary Table 1 [file 41597_2024_3434_MOESM1_ESM.docx]

**Supplementary Table 1. Water rights data sources by state**

| State | Dataset | Source | OriginalWater Unit Type | Date Collected | Citation |
| --- | --- | --- | --- | --- | --- |
| Arizona Points of Diversion | Statement of Claimant | <https://new.azwater.gov/gis> | AF, GAL, MI, AFY, CFS, GPA, GPD, GPM, GPW, GMO, MIA | February, 2020 | 1 |
| Arizona Points of Diversion | Surface Water Data | <https://new.azwater.gov/gis> | AF, CFT, GAL, MI, AFY, CFS, GPA, GPD, GPM, MIA | February, 2020 | 2 |
| Arizona Points of Diversion | Wells 55 Registry | <https://new.azwater.gov/gis> | CFS or GPM | February, 2020 | 3 |
| California Points of Diversion | California Integrated Water Quality System (CIWQS) Water Rights Database | <https://ciwqs.waterboards.ca.gov/ciwqs/ewrims/EWServlet?Redirect_Page=EWWaterRightPublicSearch.jsp&Purpose=getEWAppSearchPage> | AF, AFY, CFS, GPD, GPM | February, 2020 | 4 |
| Colorado Points of Diversion | Colorado Water Rights - Net Amounts | <https://dwr.state.co.us/Tools/WaterRights/NetAmounts> | CFS | February, 2020 | 5 |
| Idaho Points of Diversion | Point of Diversion: Water Right | https://data-idwr.hub.arcgis.com/datasets/IDWR::water-right-pods/ | CFS | May, 2017 | 6 |
| Idaho Places of Use | Place of Use: Water Right | https://data-idwr.hub.arcgis.com/documents/IDWR::place-of-use-water-right/about | - | May, 2017 | 7 |
| Montana Points of Diversion and Places of Use | Montana Water Rights | [https://mslservices.mt.gov/Geographic_Information/Data/DataList/datalist_Details.aspx?did={0303D17E-BD0F-4180-A345-359C61E586F0}](https://mslservices.mt.gov/Geographic_Information/Data/DataList/datalist_Details.aspx?did=%7B0303D17E-BD0F-4180-A345-359C61E586F0%7D) | CFS or GPM | April, 2020 | 8 |
| Nevada Points of Diversion | Points of Diversion | <https://ndwr.maps.arcgis.com/apps/mapviewer/index.html?layers=b470345a537e4126a26154659a72a1d8> | CFS | April, 2020 | 9 |
| Nevada Places of Use | Points of Use | <https://ndwr.maps.arcgis.com/apps/mapviewer/index.html?layers=9ac96bad8ed640faa1fda409ac275589> | - | April, 2020 | 10 |
| New Mexico Points of Diversion | New Mexico Office of the State Engineer (OSE) Points of Diversion | <https://geospatialdata-ose.opendata.arcgis.com/datasets/ose-pods/explore?location=34.180669%2C-106.129109%2C7.51> | AF | April, 2020 | 11 |
| Oregon Points of Diversion and Places of Use | Statewide Water Right Spatial Data with Metadata | <https://www.oregon.gov/OWRD/access_Data/Pages/Data.aspx> | CFS | February, 2020 | 12 |
| Utah Points of Diversion | Utah Points of Diversion | <https://opendata.gis.utah.gov/datasets/utahDNR::utah-points-of-diversion> | CFS | April, 2020 | 13 |
| Utah Places of Use | Utah Place of Use | <https://opendata.gis.utah.gov/datasets/utahDNR::utah-place-of-use> | - | April, 2020 | 14 |
| Washington Places of Use | Geographic Water Information System (GWIS) | <https://fortress.wa.gov/ecy/gispublic/DataDownload/wr/GWIS_Data/> | CFS or GPM | January, 2020 | 15 |
| Wyoming Points of Diversion | Wyoming e-Permit Database | <http://seoweb.wyo.gov/e-Permit/Common/Login.aspx> | CFS or GPM | May, 2020 | 16 |

AF - Acre-feet, as a volume. Also stated as Acre-feet Total.

CFT - Cubic Feet, as a volume.

GAL - Gallons, as a volume.

MI - Miner’s Inches, as a volume. Also stated as Miner’s Inches Total.

AFY - Acre-feet Per Year. Also stated as Acre-feet Per Annum.

CFS - Cubic Feet Per Second

GMO - Gallons Per Month

GPA - Gallons Per Acre Per Year

GPD - Gallons Per Day

GPM - Gallons Per Minute

GPW - Gallons Per Week

MIA - Miner’s Inch Per Annum

### **References**

1. Arizona Department of Water Resources. Statement of Claimant. Interactive Maps & Data <https://new.azwater.gov/gis> (2020).

2. Arizona Department of Water Resources. Surface Water Data. Interactive Maps & Data <https://new.azwater.gov/gis> (2020).

3. Arizona Department of Water Resources. Wells 55 Registry. Interactive Maps & Data <https://new.azwater.gov/gis> (2020).

4. California State Water Control Board. Electronic Water Rights Information Management System (eWRIMS). California Integrated Water Quality System (CIWQS) <https://ciwqs.waterboards.ca.gov/ciwqs/ewrims/EWServlet?Redirect_Page=EWWaterRightPublicSearch.jsp&Purpose=getEWAppSearchPage> (2020).

5. Colorado Division of Water Resources. Water Rights – Net Amounts. Colorado’s Decision Support Systems <https://dwr.state.co.us/Tools/WaterRights/NetAmounts> (2020).

6. Idaho Department of Water Resources. Water Right PODs. Idaho Department of Water Resources Map & GIS Data Hub https://data-idwr.hub.arcgis.com/datasets/IDWR::water-right-pods/ (2017).

7. Idaho Department of Water Resources. Place of Use: Water Right. Idaho Department of Water Resources Map & GIS Data Hub https://data-idwr.hub.arcgis.com/documents/IDWR::place-of-use-water-right/about/ (2017).

8. Montana Water Resources Division. Montana Water Rights. Montana State Library [https://mslservices.mt.gov/Geographic_Information/Data/DataList/datalist_Details.aspx?did={0303D17E-BD0F-4180-A345-359C61E586F0}](https://mslservices.mt.gov/Geographic_Information/Data/DataList/datalist_Details.aspx?did=%7b0303D17E-BD0F-4180-A345-359C61E586F0%7d) (2020).

9. State of Nevada Division of Water Resources. Water Rights Points of Diversion. NDWR Open Data <https://ndwr.maps.arcgis.com/apps/mapviewer/index.html?layers=b470345a537e4126a26154659a72a1d8> (2020).

10. State of Nevada Division of Water Resources. Water Rights Places of Use. NDWR Open Data <https://ndwr.maps.arcgis.com/apps/mapviewer/index.html?layers=9ac96bad8ed640faa1fda409ac275589> (2020).

11. New Mexico Office of the State Engineer. OSE Points of Diversion. NM OSE Open Data Site <https://geospatialdata-ose.opendata.arcgis.com/datasets/ose-pods/explore?location=34.180669%2C-106.129109%2C7.51> (2020).

12. Oregon Water Resources Department. Water Rights Information System (WRIS). Water Right Research Query <https://www.oregon.gov/OWRD/access_Data/Pages/Data.aspx> (2020).

13. Utah Department of Natural Resources. Utah Points of Diversion. Utah’s State Geographic Information Database <https://opendata.gis.utah.gov/datasets/utahDNR::utah-points-of-diversion> (2020).

14. Utah Department of Natural Resources. Utah Place of Use. Utah’s State Geographic Information Database <https://opendata.gis.utah.gov/datasets/utahDNR::utah-place-of-use>  (2020).

15. State of Washington Department of Ecology. Water Rights Places of Use. Geographic Water Information System Database <https://ecology.wa.gov/Research-Data/Data-resources/Geographic-Information-Systems-GIS/Data> (2020).

16. Wyoming State Engineer’s Office. Water Rights. e-Permit Database <http://seoweb.wyo.gov/e-Permit/Common/Login.aspx> (2020).
