# Supplementary material for: Harmonized Database of Western U.S. Water Rights (HarDWR) v.1
Source: Sci Data. 2024 Jun 6;11:598. doi: 10.1038/s41597-024-03434-6 (PMC11156903; doi:10.1038/s41597-024-03434-6)
Supplement: Supplementary file 2 — Supplementary Table 2 [file 41597_2024_3434_MOESM2_ESM.docx]

**Supplementary Table 2. Water Management Area (WMA) spatial boundary data sources by state**

| State | Dataset | Source | Date Collected | Citation |
| --- | --- | --- | --- | --- |
| Arizona Surface Water | Surface Watershed | <https://gisdata2016-11-18t150447874z-azwater.opendata.arcgis.com/datasets/surface-watershed/explore?location=34.158174%2C-111.970823%2C7.50> | February, 2020 | 1 |
| Arizona Ground Water | Groundwater Basin | <https://gisdata2016-11-18t150447874z-azwater.opendata.arcgis.com/datasets/groundwater-basin-2/explore?location=34.158174%2C-111.970823%2C7.50> | February, 2020 | 2 |
| California | CalWater 2.2.1 | <https://www.mlml.calstate.edu/mpsl-mlml/data-center/data-entry-tools/data-tools/gis-shapefile-layers/> | February, 2020 | 3 |
| Colorado | Colorado Water District Boundaries | <https://www.colorado.gov/pacific/cdss/gis-data-category> | February, 2020 | 4 |
| Idaho | Idaho Department of Water Resources (IDWR) Administrative Basins | https://data-idwr.hub.arcgis.com/datasets/idwr-administrative-basins/explore | November, 2015 | 5 |
| Montana |  | Directly contacted Montana Department of Natural Resources and Conservation (DNRC) Office of Information Technology (OIT) | June, 2019 | 6 |
| Nevada | State Engineer Admin Basin Boundaries | <https://ndwr.maps.arcgis.com/apps/mapviewer/index.html?layers=1364d0c3a0284fa1bcd90f952b2b9f1c> | April, 2020 | 7 |
| New Mexico | New Mexico Office of the State Engineer (OSE) Declared Groundwater Basins | <https://geospatialdata-ose.opendata.arcgis.com/datasets/ose-declared-groundwater-basins/explore?location=34.179783%2C-105.996542%2C7.51> | April, 2020 | 8 |
| Oregon | Oregon Water Resources Department (OWRD) Administrative Basins | <https://www.oregon.gov/OWRD/access_Data/Pages/Data.aspx> | February, 2020 | 9 |
| Utah | Utah Adjudication Books | <https://opendata.gis.utah.gov/datasets/utahDNR::utah-adjudication-books/explore?location=39.497165%2C-111.587782%2C-1.00> | April, 2020 | 10 |
| Washington | Water Resource Inventory Areas (WRIA) | <https://ecology.wa.gov/Research-Data/Data-resources/Geographic-Information-Systems-GIS/Data> | June, 2017 | 11 |
| Wyoming | Wyoming State Engineer’s Office Board of Control Water Districts | Directly contacted Wyoming State Engineer’s Office | June, 2019 | 12 |

### **References**

1. Arizona Department of Water Resources. Surface Watershed. Arizona Water Resources Data <https://gisdata2016-11-18t150447874z-azwater.opendata.arcgis.com/datasets/surface-watershed/explore?location=34.158174%2C-111.970823%2C7.50> (2020).

2. Arizona Department of Water Resources. Groundwater Basin. Arizona Water Resources Data <https://gisdata2016-11-18t150447874z-azwater.opendata.arcgis.com/datasets/groundwater-basin-2/explore?location=34.158174%2C-111.970823%2C7.50> (2020).

3. California Natural Resources Agency. CalWater 2.2.1. San Jose State University Marine Pollution Studies Laboratory Data Center <https://www.mlml.calstate.edu/mpsl-mlml/data-center/data-entry-tools/data-tools/gis-shapefile-layers/> (2004).

4. Colorado Division of Water Resources. District Boundaries. Colorado’s Decision Support Systems <https://www.colorado.gov/pacific/cdss/gis-data-category> (2020).

5. Idaho Department of Water Resources. IDWR Administrative Basins. Idaho Department of Water Resources Map & GIS Data Hub https://data-idwr.hub.arcgis.com/datasets/idwr-administrative-basins/explore (2019).

6. Montana DNCR Office of Information Technology. Water Administration Basins. (2019).

7. State of Nevada Division of Water Resources. State Engineer Admin Basin Boundaries. NDWR Open Data <https://ndwr.maps.arcgis.com/apps/mapviewer/index.html?layers=b470345a537e4126a26154659a72a1d8> (2020).

8. New Mexico Office of the State Engineer. OSE Declared Groundwater Basins. NM OSE Open Data Site <https://geospatialdata-ose.opendata.arcgis.com/datasets/ose-declared-groundwater-basins/explore?location=34.179783%2C-105.996542%2C7.51>  (2014).

9. Oregon Water Resources Department. WRD Administrative Basins. State Water Right Spatial Data <https://www.oregon.gov/OWRD/access_Data/Pages/Data.aspx> (2020).

10. Utah Department of Natural Resources. Utah Adjudication Books. Utah’s State Geographic Information Database <https://opendata.gis.utah.gov/datasets/utahDNR::utah-adjudication-books/explore?location=39.497165%2C-111.587782%2C-1.00>  (2019).

11. State of Washington Department of Ecology. Water Resources Inventory Areas. Geographic Water Information System Database <https://ecology.wa.gov/Research-Data/Data-resources/Geographic-Information-Systems-GIS/Data> (2020).

12. Wyoming State Engineer’s Office. Board of Control Water Districts. (2020).
