# Supplementary material for: Harmonized Database of Western U.S. Water Rights (HarDWR) v.1
Source: Sci Data. 2024 Jun 6;11:598. doi: 10.1038/s41597-024-03434-6 (PMC11156903; doi:10.1038/s41597-024-03434-6)
Supplement: Supplementary file 3 — Supplementary Table 3 [file 41597_2024_3434_MOESM3_ESM.docx]

Supplemental Table 3. Water use categories

| **Broad category** | **All character strings grouped into the broad category** |
| --- | --- |
| Irrigation | IRRIGATION, IRRIGATION 1, IRRIGATION 2, IRRIGATION 3,  IRRIGATION 4, IRRIGATION USE, DRAINAGE, Irrigation,  AGRICULTURAL SPRAYING, Agriculture other than irrigation,  IRRIGATION-CAREY ACT, IRRIGATION-DLE, Agriculture,  Supplemental Flood Harvesting,  Harvesting of Cranberries, Cranberries, Greenhouse, Mint Still,  Nursery Uses, Temperature Control, Irrigation of Cranberries,  Irrigation of Livestock and Domestic,  Primary and Supplemental Irrigation, Irrigation and Domestic,  Irrigation and Livestock, Supplemental Irrigation Non Irrigable,  Practicably Irrigable, Subsurface Irrigation,  Irrigation (includes cranberry farming, lawn/garden watering with definite acreage golf courses greenhouses, etc.),  Heat Protection For Crops (Water used during the summer months to protect such crops as apples and cranberries from the heat),  Irrigation-- Ground water,  Large scale landscape/lawn watering greater than 1 acre (golf courses cemeteries recreation areas),  Irrigation-- Surface water, Tree Watering (non-commercial) |
| Domestic | MUNICIPAL, DOMESTIC, DOMESTIC 1, DOMESTIC 10, DOMESTIC 2, DOMESTIC 3, DOMESTIC 4, DOMESTIC 5, DOMESTIC 6,  DOMESTIC 7, DOMESTIC 8, DOMESTIC 9, MUNICIPAL USE, MUNICIPAL USES, SUBDIVISION, Domestic, Municipal, Household only use, INSTITUTIONAL,  LAWN AND GARDEN, MULTIPLE DOMESTIC, Cemetery, Domestic construction, 72-12-1 domestic one household, Community type use – mdwca,  private or commercial supplied, Mobile home parks,  72-12-1 multiple domestic households, Municipal - city or county supplied water, Non 72-12-1 domestic one household,  Non 72-12-1 multiple domestic households, School use – public,  private parochial & universities, Subdivision, QUASI-MUNICIPAL,  Domestic including lawn and garden,  Domestic expanded included non-commercial gardens,  Domestic and Livestock, Group Domestic, Human Consumption,  Human Consumption and Livestock, Restroom, School, Campsite, Swimming 0, Quasi-Municipal, Air Conditioning or Heating,  Domestic General (use of water for all domestic uses not specifically defined in the water right record or not defined by the other specific domestic use categories. Includes sewage treatment farm supply and laboratory use),  Domestic Multiple (more than one dwelling i.e. motels trailer courts campgrounds parks schools port districts public utility districts diking and drainage districts water districts reclamation districs and counties none of which are under municipal control),  Domestic Single (one dwelling with lawn and garden up to one-half acre), Municiple Intertie System,  Domestic Municipal (serves general domestic commercial and industrial needs of an incorporated municipality i.e. cities towns and outlying areas),  Domestic-- Ground water, Municipal-- Ground water,  Domestic and/or Stock >25 GPM, Subdivisions, Water Districts, Water Hauls, Domestic -- Surface water, Domestic (Phase II Award0, Domestic Supply, Municipal-- Surface water, Municipal (Emergency) |
| Livestock | STOCKPOND, STOCKPOND 1, STOCKPOND 10, STOCKPOND 11, STOCKPOND 12, STOCKPOND 13, STOCKPOND 14, STOCKPOND 15, STOCKPOND 16, STOCKPOND 17, STOCKPOND 18, STOCKPOND 2, STOCKPOND 3, STOCKPOND 4, STOCKPOND 5, STOCKPOND 6, STOCKPOND 7, STOCKPOND 8, STOCKPOND 9,  STOCKWATERING OTHER THAN FROM A STOCKPOND, STOCK, COWS / HORSES, Stockwatering, Stock, Dairy operation, 72-12-1 domestic and livestock watering,  Feed pen operation, Non 72-12-1 domestic and livestock watering,  Non 72-12-1 livestock watering, Poultry and egg operation,  72-12-1 livestock watering, STOCKWATERING, Dairy Barn Livestock,  Livestock and Wildlife Dairy Stock Water (includes domestic uses of water for dairy/cattle farms game bird farming poultry farming and fur-bearing animal farming),  Stock Watering, Commercial Agriculture (feedlots confined swine dairies),  Stock Water Pipelines, Stockyard (Pig Cow Chicken), Stock and/or Domestic,  Stock (Aesthetics), Stock and Domestic |
| Fish | Fish and Wildlife Preservation and Enhancement, Fishery, FISH AND WILDLIFE, FISH RACEWAYS, FISHERY, FLOW THROUGH FISH POND, INSTREAM FISHERY, Fish and game propogation, Aquaculture, Fish Culture, Fish and Wildlife, Supporting Aquatic Life, Anadromous and Resident Fish Habitat,  Instream Fishery Enhancement,  Fish Propagation (includes water service to ponds reservoirs hatcheries and all other facilities involved in the overall purpose of fish propagation),  Fish Propagation, Fish Propagation (Aesthetics) |
| Industrial | COMMERICAL MINING, INDUSTRIAL/COMMERCIAL USE, MINING USE, POWER USE, POWER, INDUSTRIAL,  COMMERCIAL UTILITY (WATER CO) OTHER - MINERAL EXPLORE,  OTHER - PRODUCTION , RECOVERY TEST, DEWATERING, REMEDIATION, Heat Control, Incidental Power, Industrial, Milling, Mining, Power, Snow Making, Commercial, Evaporative, Geothermal, Power Generation, GEOTHERMAL HEATING, GEOTHERMAL OBSERVATION AND TESTING, OIL WELL FLOODING, POLLUTION ABATEMENT, POWER GENERATION, POWER GENERATION NONCONSUMPTIVE, SEDIMENT CONTROL, Brine production, well Construction, Cathodic protection well, Dewatering well, Exploration, Geothermal boreholes, Highway construction Injection, Manufacturing, Military - military installations, Mining or milling or oil, Meat packing plant, Oil field maintenance, Oil production, Pollution control well, Petroleum processing plant,  72-12-1 Prospecting or development of natural resource,  72-12-1 Construction of public works,  72-12-1 Sanitary in conjunction with a commercial use,  Secondary recovery of oil, Public utility, CONSTRUCTION, MINING AND MILLING, MINING, MILLING AND DEWATERING EVAPORATION, Commercial Uses,  General Construction or Maintenance, Geo-Thermal (Heating and Cooling), Laboratory, Log Deck Sprinkling, Shop, Sawmill, Geo-Thermal (Energy Production), Power Development Ram, Pollution Abatement, Forest Management,  Road Construction, Cooling for industrial purposes,  Commercial and Industrial Manufacturing (includes food processing and packaging sand and gravel processing asphalt plant metal processing and manufacturing pulp and paper manufacturing aquatic plant culture petroleum refining car washes and laundries),  Mining (includes washing coal dredge mining and hydraulic mining),  Railway (use of water to serve railway equipment and facilities),  Power (includes hydro-electric hydraulic ram and thermo-electic),  Heat Exchange (use of such equipment as heat pumps refrigeration equipment and other cooling devices),  Coal Bed Methane-- Ground water, Industrial-- Ground water, Test Well,  Mine Dewatering, Highway Construction (temporary), Oil and Gas Well Drilling, Coal Bed, Natural Gas, Hydropower, Ice Cutting, Industrial-- Surface water, Railroad, Transportation, Utilities, Coal Bed Natural Gas (Aesthetics),  Bottling Water, Chemical, Culinary, Maintenance (Equipment Washing), Mechanical, Medicinal, Other - Commercial, Dewatering, Drilling, Heat Extraction, Other -- Industrial, Pollution Control, Refining, Reclamation Watering,  Sediment Control, Dust Abatement, Hydrostatic Testing, Well Drilling |
| Environmental | RECREATION RECREATION USE, WILDLIFE, MONITORING, Aquaculture,  Dust Control, Recreational, Water Quality Storage, Recreation, Augmentation, Cumulative Accretion to River, Cumulative Depletion from River,  Federal Reserved, Minimum Streamflow Net Effect on River, Recharge, Wildlife, AQUIFER RECHARGE, AUGMENTATION, EROSION CONTROL, FLOOD CONTROL, INSTREAM FLOW, MARKETING FOR MITIGATION/AQUIFER RECHARGE, MITIGATION WATER, STORAGE, WATERFOWL WETLAND, WETLAND MITIGATION CREDIT, WILDLIFE HABITAT,  WILDLIFE HABITAT MAINTENANCE AND ENHANCEMENT, WILDLIFE/WATERFOWL, Flood control, Monitoring well, Observation, Strategic water reserve, ENVIRONMENTAL, Multiple Instream Uses, Flow Augmentation, Fish Enhancement, Instream Fire Protection, Riparian Habitat, Aesthetics, Groundwater Recharge, Groundwater Aquifer Storage and Recovery,  Pond Maintenance, Riparian Habitat Restoration Project,  Storage (Storage of water),  Groundwater Preservation (essentially a groundwater trust right),  Environmental Quality (includes pollution control dust control flood control or any water use which improves or maintains the quality of the environment), Instream flow, Trust Water-Permanent (Water in permanent trust),  Trust Water-Temporary (Water in temporary trust),  Wildlife Propagation (includes water to service non-domesticated animals such as birds game and non-game species),  Recreation and Beautification (includes beautifying private and public grounds and supplying water to swimming pools boating ponds etc),  Monitor, Observation, Wetlands, Consumptive Instream Flow,  Maintain Natural Condition (Phase II Award), Erosion Control, Flood Control, Flow Through, Ground Water Recharge, Instream Flow (Phase II Award),  Instream Flow- only State of Wyo can apply,  Maintain Natural Lake Level (Phase II Award), Natural Flow (Phase II Award), Stream Wild and Scenic-only State of Wyo can apply,  Ground Water Recharge (Aesthetics), Recreation (Aesthetics),  Wetlands (Aesthetics), Wildlife (Aesthetics) |
| Other | OTHER USES, NO USE CLAIMED, OTHER USES 1, OTHER USES 10, OTHER USES 11, OTHER USES 12, OTHER USES 13, OTHER USES 14, OTHER USES 15,  OTHER USES 16, OTHER USES 17, OTHER USES 2, OTHER USES 3, OTHER USES 4, OTHER USES 5, OTHER USES 6, OTHER USES 7, OTHER USES 8, OTHER USES 9, LOST, OTHER None Stated, NO USE CODE ON NOI, NO WATER USE, RESERVED, UNKNOWN, Aestetic, Fire Protection, Frost Protection, Other, Fire, Export from Basin, Export from State, Transmountain Export, All Beneficial Uses,  FIRE PROTECTION, NAVIGATION, OTHER PURPOSE, SALE, WATER MARKETING, UNKONWN, Augmentation well, Closed file, No pre-1907 water right exists on this land, Non-profit organizational use, No use of right or POD, No right,  Stacked water right, To be determined, AS DECREED, RECREATIONAL, Recreation, Multiple Purpose, Pollution Abatement,  Frost Protection (frost protection other than cranberries),  Fire Protection (includes sprinkling log storage facilities),  No purpose Identified Other (No purpose identified), Unknown,  Miscellaneous-- Ground water, Others -- Miscellaneous, Reservoir Supply, Combined Uses, Existing Capacity, Temporary, Total Enlargement for this application, Other -- Temporary, Bathing |
